# Success of Deuruxolitinib (Leqselvi™) in Extensively Treatment-Resistant Alopecia Universalis: A Case Report

**DOI:** 10.7759/cureus.111710

**Published:** 2026-06-29

**Authors:** Simona Kogan, Moises Lutwak, Daniel Lutwak, Tara Howard, Stanley Skopit, Sumeet Bhardwaj

**Affiliations:** 1 Dermatology, Philadelphia College of Osteopathic Medicine (PCOM), Philadelphia, USA; 2 Dermatology, Larkin Community Hospital, South Miami, USA; 3 Dermatology, Dania Dermatology, Dania, USA; 4 Psychiatry, Kansas City University, Kansas City, USA

**Keywords:** alopecia areata universalis, autoimmunity, biologics, leqselvi, treatment resistant

## Abstract

Alopecia universalis (AU) is a severe autoimmune disorder characterized by complete loss of scalp and body hair. The introduction of Janus kinase (JAK) inhibitors has expanded therapeutic options for patients with severe disease; however, treatment responses remain variable, particularly among individuals with extensive and longstanding hair loss. Reporting cases with atypical or limited responses to therapy remains important for understanding the spectrum of clinical outcomes observed in practice.

We describe a 30-year-old man with a 23-year history of AU who demonstrated minimal response to multiple prior therapies, including tofacitinib (pan-JAK inhibitor) and ritlecitinib (JAK3/TEC inhibitor), without meaningful scalp, eyebrow, or eyelash regrowth. Following six months of treatment with deuruxolitinib, the patient experienced his first clinically significant regrowth, limited to the eyebrows and eyelashes, while scalp involvement remained largely unchanged. This case highlights the potential for site-specific hair regrowth in longstanding, treatment-refractory AU and suggests that eyebrow and eyelash regrowth may occur even in the absence of substantial scalp hair recovery.

## Introduction

Alopecia areata (AA) is an autoimmune disorder characterized by loss of hair follicle immune privilege, primarily mediated by interferon-gamma (IFN-γ)-activated CD8+ NKG2D+ T cells. Janus kinase (JAK) inhibitors have revolutionized treatment options for moderate-to-severe AA through inhibition of the IFN-γ signaling pathway [1-3]. Deuruxolitinib is an oral, selective Janus kinase (JAK) 1/2 inhibitor that suppresses the JAK-STAT signaling pathway, thereby reducing the CD8+ T-cell-mediated autoimmune attack responsible for hair follicle destruction in alopecia areata. It was approved in July 2024 for the treatment of severe alopecia areata in adults [4].

The clinical presentation of alopecia areata (AA) exists along a spectrum of disease extent and pattern. Patchy AA is the most common presentation and is characterized by well-circumscribed, nonscarring patches of hair loss. Alopecia totalis (AT) involves the complete loss of scalp hair, whereas alopecia universalis (AU), as seen in our patient, represents the most extensive form, with complete loss of scalp and body hair [5]. Several pattern-specific variants have also been described. Ophiasis presents as band-like hair loss involving the occipital and temporal scalp margins and is often associated with a chronic, treatment-resistant course and poorer prognosis [6]. Sisaipho (ophiasis inversus) is the reverse pattern, characterized by frontal and parietal hair loss with sparing of the occipital scalp. Diffuse AA causes generalized scalp thinning without distinct patches and may mimic other causes of nonscarring alopecia [6]. Less common variants include alopecia areata reticularis, which demonstrates a net-like pattern of hair loss, and perinevoid alopecia areata, characterized by localized alopecia surrounding a melanocytic nevus [7]. Additional subtypes include acute diffuse and total alopecia (ADTA), a rapidly progressive form characterized by diffuse shedding that may progress to extensive hair loss but often carries a more favorable prognosis than classic AT or AU, and canities subita ("sudden graying"), in which selective loss of pigmented hairs leaves preexisting gray or white hairs intact, creating the appearance of abrupt hair whitening [8]. These diverse presentations highlight the broad clinical spectrum of AA.

Disease severity is an important prognostic factor in AA. In the TOAST classification system (Topography-based Alopecia areata Severity Tool), patients with limited hair loss (Grade 1) demonstrated a major regrowth rate of 93.4%, compared with only 28.2% among those with near-total hair loss (Grade 4). Additional poor prognostic factors include alopecia totalis or universalis, nail involvement, concomitant autoimmune disease, early age of onset, a positive diffuse hair-pull test, and rapid disease progression [9].

Consistent with these observations, clinical trials including THRIVE-AA1/2 (deuruxolitinib) and ALLEGRO (ritlecitinib) demonstrate variable treatment responses, with patients who have longstanding disease (>5-10 years) and baseline Severity of Alopecia Tool (SALT) scores of 100, consistent with alopecia universalis (AU), being less likely to achieve the primary endpoint of SALT ≤20 [10]. We present a case of a 30-year-old man with a 23-year history of AU who demonstrated primary nonresponse to sequential treatment with tofacitinib and ritlecitinib, but subsequently developed eyebrow and eyelash regrowth following treatment with deuruxolitinib. This case is notable because it demonstrates a partial clinical response to deuruxolitinib in a patient with longstanding, treatment-refractory AU and multiple adverse prognostic factors, suggesting that therapeutic benefit may still be achievable in select patients despite prior failure of other JAK inhibitors.

## Case presentation

A 30-year-old man presented with treatment-refractory alopecia universalis (AU) with a 23-year disease history and a baseline SALT score of 100. He was initially diagnosed with alopecia areata at age 7, which rapidly progressed to complete scalp and body hair loss consistent with AU. His disease course was later accompanied by Onychorrhexis. Past medical history was significant for hypothyroidism and atopic comorbidities including eczema, asthma, and allergic rhinitis, which may reflect overlapping inflammatory pathways beyond isolated IFN-γ signaling.

The patient had failed multiple prior therapies. Topical minoxidil 5% foam in combination with clobetasol propionate 0.05% did not produce clinically significant regrowth (Table 1). Short-contact anthralin 0.5-1% cream was also ineffective (Table 1). Treatment with squaric acid dibutyl ester (SADBE) immunotherapy was discontinued because of severe local adverse reactions (Table 1). Systemic corticosteroids were deferred due to concerns regarding long-term adverse effects (Table 1).

**Table 1:** Summary of Standard Therapies and Investigational JAK Inhibitors for Alopecia Universalis Table adapted from reference [4].

Medication	Mechanism of Action	Standard Dosing	FDA approval	Patient Dosing	Duration	Clinical response
Clobetasol propionate 0.05% (topical)	High potency	Apply thin film BID; typical course 6–12 weeks, extendable to 3–6 months	Not FDA approved	BID	3-6 months	No meaningful hair growth; discontinued
Anthralin 0.5–1% (short-contact topical)	Irritant; induces local inflammation	0.5–1% cream applied daily for 20–60 min then rinsed	Not FDA approved	Daily (20–60 min)	6 months	No regrowth; discontinued
SADBE (Squaric acid dibutyl ester, topical immunotherapy)	Contact immunotherapy induces a local allergic reaction	Sensitization with 2% once, then 0.001–2% weekly under occlusion 24–48h	Not FDA approved	0.001% → titrated to 2% weekly under occlusion	>6 months	No regrowth; blistering, pruritus, pigment changes → discontinued
Minoxidil 5% foam (topical, OTC)	Vasodilator; prolongs anagen phase	1 mL BID to the affected scalp	FDA approval for androgenetic alopecia	BID	6 months	No response
Tofacitinib (oral)	JAK1/3 inhibitor	5 mg BID; escalation to 10 mg BID in non-responders (off-label)	Not FDA approved	5 mg BID	6 months	No regrowth
Ritlecitinib (oral, Litfulo®)	JAK3/TEC inhibitor	50 mg QD consensus	FDA-approved	50 mg QD	6 months	No regrowth; novel AE: episodic asymmetric neuropathic-like musculoskeletal pain; discontinued
Deuruxolitinib (oral, Leqselvi®)	JAK1/2 inhibitor	8–12 mg BID investigational	FDA-approved	Adjusted for CYP2C9	6 months	Eyelash growth; discontinued

**Figure 1:**
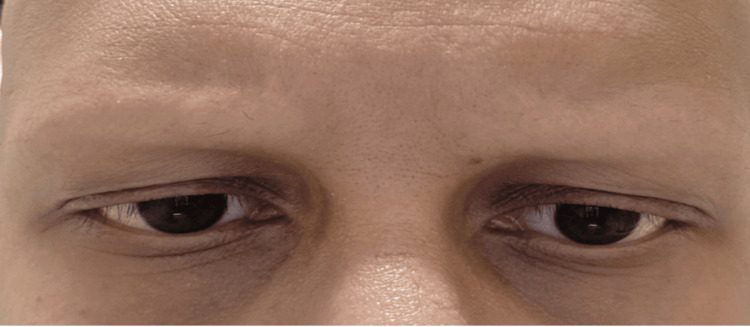
Baseline before initiation of deuruxolitinib

**Figure 2:**
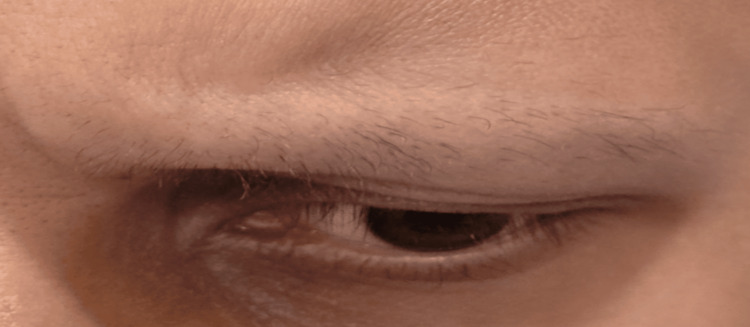
After the initial two-month trial of deuruxolitinib Eyebrow and eyelash growth happened separately from scalp terminal hair growth.

The patient subsequently underwent sequential treatment with multiple JAK kinase inhibitors, including tofacitinib and ritlecitinib, without meaningful scalp, eyebrow, or eyelash regrowth (Table 1, Figure 1). The patient was found to be an intermediate CYP2C9 metabolizer and had to get pretreatment evaluation prior to initiating deuruxolitinib 8 mg twice daily. The patient developed noticeable eyebrow regrowth within two months, representing the first clinically significant hair regrowth observed during his disease course despite longstanding treatment resistance (Table 1, Figure 2). Eyebrow regrowth was assessed using a retrospective application of the Brigham Eyebrow Tool for Alopecia (BETA). The patient had complete (100%) eyebrow hair loss at baseline, corresponding to an estimated maximum BETA score of 16. Follow-up photographs demonstrated approximately 75% regrowth, with an estimated BETA score of 3-4, consistent with clinically meaningful improvement [9]. In follow-up, the patient remained on deuruxolitinib for six months and demonstrated continued improvement in eyebrow and eyelash growth. Despite this partial response, therapy was discontinued because no clinically significant scalp hair regrowth occurred, and the patient's SALT score remained 100, indicating persistent alopecia universalis.

Disclaimer: BETA score was assessed using the formula (surface area × density) and should be interpreted as approximate rather than formal validated assessments [9].

## Discussion

The case provides valuable practical clinical information on the management of severe, chronic AU. The results of the THRIVE-AA1/AA2 trials showed that patients achieved SALT ≤20 (≥80% scalp hair coverage) at Week 24 [10]. However, patients with more severe disease (baseline SALT ≥95, meaning alopecia universalis/near universalis) responded less well overall, with only 20.9-26.0% of achieved SALT ≤20 at Week 24. The subsequent development of clinically appreciable eyebrow regrowth following initiation of deuruxolitinib may suggest that selective JAK1/2 inhibition retains therapeutic activity in certain patients with longstanding, treatment-refractory AU despite failure of alternative JAK inhibitor subclasses [10]. In one study from a systematic review and Bayesian network meta-analysis of seven randomized controlled trials comparing approved oral JAK inhibitors for severe alopecia areata suggested that deuruxolitinib 8 mg twice daily may have the highest short-term efficacy at 24 weeks, demonstrating superior outcomes to baricitinib and numerically favorable outcomes compared with ritlecitinib in indirect analyses [11]. Another study evaluating deuruxolitinib found that 41.3% of patients treated with 12 mg twice daily achieved SALT ≤20 after 24 weeks, significantly outperforming placebo (0.8%) and demonstrating greater efficacy than that reported for ritlecitinib 50 mg and baricitinib 4 mg in severe alopecia areata [12]. Stronger suppression of IFN-γ-driven inflammation through JAK1/2 inhibition may explain the partial clinical response observed in our patient (Table 2).

**Table 2:** Comparison of FDA-approved Janus kinase (JAK) inhibitors for alopecia areata. Table adapted from reference [5].

Feature	Baricitinib (Olumiant)	Ritlecitinib (Litfulo)	Deuruxolitinib (Leqselvi)
JAK Selectivity	JAK1/JAK2	JAK3/TEC kinase family	JAK1/JAK2
Approved Dose	2 mg or 4 mg once daily	50 mg once daily (optional 200 mg loading dose × 4 weeks)	8 mg twice daily
Age Indication	Adults (≥18 years)	Adults and adolescents (≥12 years)	Adults (≥18 years)
Efficacy	SALT ≤20 at Week 36	SALT ≤20 at Week 24	SALT ≤20 at Week 24
Key Adverse Events	Acne, ↑CK, ↑LDL/HDL, URI, UTI, herpes zoster	Headache, URI, pyrexia, herpes zoster	↑CK, headache, acne (dose-dependent)

Now let's examine the safety profile of JAK inhibitors:

The ORAL Surveillance Trial and Its Limitations for the AA/AU Population (Alopecia Areata/Alopecia Universalis)

The FDA boxed warning applied to all JAK inhibitors including deuruxolitinib was precipitated by the ORAL Surveillance trial, which enrolled 4,362 patients with rheumatoid arthritis aged ≥50 years with at least one additional cardiovascular risk factor, and found that tofacitinib was associated with higher rates of MACE or major adverse cardiovascular events (HR 1.33, 95% CI 0.91-1.94) and malignancy (HR 1.48, 95% CI 1.04-2.09) compared to TNF inhibitors over a median 4-year follow-up [12].

Critically, this population differs fundamentally from the AA/AU cohort in several respects. The mean age in the ORAL Surveillance trial was 61 years, compared to approximately 38-39 years in AA clinical trial populations and epidemiologic cohorts [13-14].

Patients in ORAL Surveillance had prevalent cardiovascular risk factors by design and were receiving concomitant methotrexate and often systemic corticosteroids unlike AA/AU patients, who typically receive JAK inhibitor monotherapy and carry a low baseline Charlson Comorbidity Index (mean 0.1) [15].

These demographic and comorbidity differences likely explain why two independent meta-analyses of JAK inhibitors used specifically for dermatologic indications encompassing over 20,000 and 12,000 patients, respectively, found no significant increase in composite MACE/all-cause mortality or VTE compared to placebo [10,15].

A large multinational real-world cohort study (17,068 propensity-matched patients with skin IMIDs) further corroborated these findings, demonstrating that JAK inhibitors were associated with lower incidences of all-cause mortality (HR 0.47, 95% CI 0.25-0.88) and MACE (HR 0.63, 95% CI 0.46-0.88) compared to conventional immunomodulators over two years, with no increased VTE or malignancy risk even in older adults and those with cardiometabolic risk factors [10].

What we saw with the phase 3 THRIVE-AA2 trial showed that deuruxolitinib was generally well tolerated in the phase 3 THRIVE-AA2 trial. Treatment-emergent adverse events (TEAEs) occurred in 80.5% of patients receiving 8 mg twice daily and 81.4% receiving 12 mg twice daily, compared with 70.0% in the placebo group [16]. Most adverse events were mild to moderate in severity. The most common adverse events included COVID-19 infection, nasopharyngitis, headache, acne vulgaris, and elevations in creatine phosphokinase levels [16]. Serious adverse events were uncommon, occurring in 1.2% and 1.6% of patients receiving 8 mg and 12 mg twice daily, respectively. Treatment discontinuation due to adverse events was infrequent (3.1% and 2.3%, respectively) [16].

In summary, this case demonstrates that deuruxolitinib may provide clinical benefit in select patients with longstanding, treatment-refractory alopecia universalis, even after failure of other JAK inhibitors. Although deuruxolitinib carries the FDA boxed warning shared by all JAK inhibitors, no deaths, myocardial infarctions, strokes, or thromboembolic events were reported in the 24-week THRIVE-AA2 trial. Importantly, the boxed warning is based primarily on the ORAL Surveillance study of tofacitinib in older patients with rheumatoid arthritis and significant cardiovascular risk factors, a population that differs substantially from the younger and generally healthier alopecia areata population [10, 16]. These findings support the continued evaluation of deuruxolitinib as a treatment option for severe alopecia universalis.

## Conclusions

This case highlights the potential utility of deuruxolitinib in patients with longstanding, treatment-refractory alopecia universalis who have failed multiple prior therapeutic modalities, including distinct JAK inhibitor subclasses. Although significant scalp regrowth was not achieved, the development of eyebrow and eyelash regrowth after years of complete hair loss suggests residual follicular responsiveness and may represent an early marker of treatment benefit. While potential confounding factors, including the natural fluctuating course of alopecia areata, delayed effects of prior therapies, and regional differences in hair follicle responsiveness, cannot be completely excluded, the timing and pattern of regrowth following initiation of deuruxolitinib support a possible therapeutic effect. Reporting such cases is important to better characterize treatment expectations in severe alopecia universalis and to improve understanding of differential responses among JAK inhibitors.
